# Incidence of multidrug-resistant organisms causing ventilator-associated pneumonia in a tertiary care hospital: A nine months' prospective study

**DOI:** 10.4103/1817-1737.32230

**Published:** 2007

**Authors:** Arindam Dey, Indira Bairy

**Affiliations:** *Department of Microbiology, Kasturba Medical College, Manipal, India*

**Keywords:** Multidrug-resistant organisms, nosocomial pneumonia, ventilator-associated pneumonia

## Abstract

**BACKGROUND:**

Ventilator-associated pneumonia (VAP) is an important intensive care unit (ICU) infection in mechanically ventilated patients. VAP occurs approximately in 9-27% of all intubated patients. Due to the increasing incidence of multidrug-resistant organisms in ICUs, early and correct diagnosis of VAP is an urgent challenge for an optimal antibiotic treatment.

**AIM OF THE STUDY:**

The aim of the study was to assess the incidence of VAP caused by multidrug-resistant organisms in the multidisciplinary intensive care unit (MICU) of our tertiary care 1,400-bedded hospital.

**MATERIALS AND METHODS:**

This prospective study was done in the period from December 2005 to August 2006, enrolling patients undergoing mechanical ventilation (MV) for >48 h. Endotracheal aspirates (ETA) were collected from patients with suspected VAP, and quantitative cultures were performed on all samples. VAP was diagnosed by the growth of pathogenic organism ≥10^5^ cfu/ml.

**RESULTS:**

Incidence of VAP was found to be 45.4% among the mechanically ventilated patients, out of which 47.7% had early-onset (<5 days MV) VAP and 52.3% had late-onset (>5 days MV) VAP. Multiresistant bacteria, mainly *Acinetobacter spp.* (47.9%) and *Pseudomonas aeruginosa (27%),* were the most commonly isolated pathogens in both types of VAP. Most of the isolates of *Escherichia coli* (80%) and *Klebsiella pneumoniae* (100%) produced extended-spectrum beta lactamases (ESBLs). As many as 30.43% isolates of *Acinetobacter spp.* showed production of AmpC beta lactamases among all types of isolates. Metallo-beta lactamases (MBLs) were produced by 50% of *Pseudomonas aeruginosa* and 21.74% of *Acinetobacter spp*.

**CONCLUSION:**

High incidence (45.4%) of VAP and the potential multidrug-resistant organisms are the real threat in our MICU. This study highlighted high incidence of VAP in our setup, emphasizing injudicious use of antimicrobial therapy. Combined approaches of rotational antibiotic therapy and education programs might be beneficial to fight against these MDR pathogens and will also help to decrease the incidence of VAP.

Ventilator-associated pneumonia (VAP), an important form of hospital-acquired pneumonia (HAP), specifically refers to pneumonia developing in a mechanically ventilated patient more than 48 h after tracheal intubation or tracheostomy. VAP requires a rapid diagnosis and initiation of the appropriate antibiotic treatment, since many studies have shown that the delayed administration of appropriate antibiotic therapy in patients with VAP has been associated with excess hospital mortality.[1] Similarly, the timely prescription of an initial antibiotic regimen that is inappropriate for the microorganism(s) causing VAP has also been associated with a significantly greater risk of death.[2,3]

VAP occurs approximately in 9–27% of all intubated patients.[4,5] Due to increasing incidence of multidrug-resistant (MDR) organisms in ICUs, early and correct diagnosis of VAP is an urgent challenge for optimal antibiotic treatment. VAP may be caused by a wide spectrum of bacterial pathogens, which may be polymicrobial and are rarely due to viral or fungal pathogens in immunocompetent hosts.[4,6,7] Common pathogens include aerobic gram-negative bacilli, such as *Pseudomonas aeruginosa*, *Escherichia coli*, *Klebsiella pneumoniae* and *Acinetobacter* species. Infections due to gram-positive cocci, such as *Staphylococcus aureus*, are more common in patients with diabetes mellitus and head trauma.[8] The frequency of specific MDR pathogens causing VAP may vary by hospital, patient population, exposure to antibiotics, type of ICU patient and changes over time, emphasizing the need for timely local surveillance data.[9]

Detection of the causative organism is crucial for the diagnosis of VAP. This is done by collecting the lower respiratory tract sample either by invasive (protected specimen brush [PSB] or broncho-alveolar lavage [BAL]) or noninvasive (endotracheal aspirate [ETA]) techniques and culturing quantitatively or semi-quantitatively. The major difficulty of this approach is in obtaining samples from the lower respiratory tract - mainly because of its probable contamination with the upper airway flora, which may result in misinterpretation of cultures.[10]

The American Thoracic Society (ATS) guidelines recommend that quantitative cultures can be performed on ETA or samples collected either bronchoscopically or nonbronchoscopically.[11] Reliance on semi-quantitative cultures, which may not reliably separate true pathogens from colonizers, can lead to either more or broader-spectrum antibiotic therapy than with a quantitative approach. On the other hand, there are many studies which compared the diagnostic value of quantitative cultures of bronchoscopic and nonbronchoscopic samples in VAP. No technique could consistently be shown to achieve a superior diagnostic yield as compared with another.[12,13] Another advantage in terms of cost – lower respiratory tract sample collection through endotracheal tube is much less expensive compared to BAL or PSB and hence is widely preferable in most of the hospital settings. The aim of our study was to assess the incidence of VAP caused by MDR organisms in the multidisciplinary intensive care unit (MICU) of our tertiary care hospital.

## Materials and Methods

This prospective study was done in the period from December 2005 to August 2006 in our hospital MICU. Patients who were receiving mechanical ventilation (MV) for >48 h were enrolled for the study. Baseline statuses of the patients were evaluated by checking body temperature, total leukocyte count, oxygenation [PaO2/FiO2 mmHg] and pulmonary radiography. Clinical pulmonary infection score (CPIS), developed by Pugin *et al.,*[14] was followed as a screening method for clinically suspected VAP.

### Collection of endotracheal aspirates (ETA)

A trained respiratory therapist collected ETA every time. The ETA was collected using a 22-inch Ramson's 12 F suction catheter with a mucus extractor, which was gently introduced through the endotracheal tube for a distance of approximately 25–26 cm. Gentle aspiration was then performed without instilling saline, and the catheter was withdrawn from the endotracheal tube. After the catheter was withdrawn, 2 ml of sterile 0.9% normal saline was injected into it with a sterile syringe to flush the exudates into a sterile container for collection. At least 0.5 ml of undiluted sample was collected in Robertson's cooked meat (RCM) anaerobic media and transported to microbiology laboratory. ETA samples were also immediately taken to the laboratory for processing. The results of the Gram's stain were obtained within the first hour and quantitative cultures were performed immediately. In patients with repeated incidence of VAP symptoms, a repeat culture was performed.

### Microbiological processing

Samples were mechanically liquefied and homogenized by vortexing for 1 min and then serially diluted in 0.9% sterile saline solution with final dilutions of 10^−2^, 10^−3^ and 10^−4^. The samples were then plated on sheep blood agar (SBA), chocolate agar (CA), MacConkey agar (MA) and Saboraud's dextrose agar (SDA) by using 4 mm Nichrome wire loop (Hi-media, Mumbai, India), which holds 0.01 ml of solution. All plates were incubated overnight at 37°C and chocolate agar plates at 37°C in 5% CO_2_ incubator, and one SDA plate was kept at room temperature. All plates were checked for growth overnight and then after 24 and 48 h of incubation. SDA plates were checked for any growth up to one week. Directly inoculated RCM bottles were first incubated for 48 h at 37°C and then cultured on Neomycin blood agar plates and incubated at 37°C in anaerobic jar for 48 h. For definite diagnosis of VAP in this study, quantitative culture threshold was considered as 10^5^ cfu/ml, as shown previously.[13,15,16] Growth of any organism below the threshold was assumed to be due to colonization or contamination. Any growth was characterized by colony morphology and Gram stain from the plates. Detailed biochemical testing identified any significant growth, and antibiotic sensitivity testing was performed on Mueller-Hinton agar (MHA) plates by Kirby-Bauer's method, and suspected extended-spectrum beta lactamases (ESBLs) producing organisms were confirmed by combination disk test as described previously.[17] Isolates showing reduced susceptibility to either ceftazidime (30 μg) or cefotaxime (30 μg) and cefoxitin (30 μg) disks were considered as ‘screen positive’ for AmpC beta lactamases and selected for detection of plasmid-mediated AmpC by the popular AmpC disk test.[18] Isolates showing reduced susceptibility to carbapenems (imipenem and meropenem) were selected for detection of metallo-beta lactamases (MBLs) enzymes by imipenem-EDTA disk method.[19]

All the statistical analysis was done by using Fisher's exact test and Mann-Whitney test.

## Results

Total 212 patients were admitted in MICU in the period from December 2005 to August 2006, and 97 patients were enrolled for the study according to the inclusion criteria. Quantitative culture results were significant (≥10^5^ cfu/ml) for pathogenic organisms causing VAP in 44 (45.4%) [*P*<0.0001 by Fisher's exact test] patients. Fifty-three (54.6%) patients did not have VAP, and they were taken as non-VAP control group. For 51 clinically suspected VAP patients, CPIS scoring was >6; and among them, by quantitative culture of ETA, 41 patients showed colony count ≥10^5^ cfu/ml. The remaining 46 patients showed CPIS <6; however, by quantitative ETA culture, 3 patients among them had pathogenic organism colony count ≥10^5^ cfu/ml. As in the present study, quantitative ETA culture was considered as the definitive standard method; therefore, those 3 cases were included within VAP group. Patients who developed VAP within 96 h of MV were categorized as having ‘early-onset VAP,’ and those who developed VAP after 96 h were classified as ‘late-onset VAP.’ Out of these 44 cases, 47.7% (21/44) were categorized under the early-onset group and the remaining 52.3% (23/44) under the late-onset group. The incidence of VAP increased with the duration of MV. The median duration of MV in non-VAP group was 5.8 days as against 19.6 days in patients with VAP (*P*<0.05) [Mann-Whitney test].

The clinical spectrums of patients shown in Table 1 indicate the highest number of patients enrolled in our study was from postoperative wards (n = 23), followed by road-traffic accident (n=16). Other conditions like leptospirosis, complicated malaria, organophosphorus poisoning cases were less in number.

**Table 1:** Clinical spectrum of patients

Disease	No. of patients (n)	VAP (%)	Non-VAP (%b)
OP poisoning	5	3 (60)	2 (40)
Road traffix accident	16	8 (50)	8 (50)
Leptospirosis	4	2 (50)	2 (50)
Malaria	3	2 (66.67)	1 (33.33)
Malignancy	11	5 (45.45)	6 (54.55)
Subdural haematoma	6	4 (66.67)	2 (33.33)
Post-operative patients	23	9 (39.13)	14 (60.87)
CRF/ARF/DM/HTN/IHD	16	7 (43.75)	9 (56.255)
Acute pancreatitis	3	1 (33.33)	2 (66.67)
FUO	3	- (0)	3 (100)
Liver abscess / cirrhosis	3	2 (66.67)	1 (33.33)
Multiple myelomal	3	1 (33.33)	2 (66.67)
Alzheimer' disease	1	- (0)	1 (100)

OP - Organophosphorus, CRF - Chronic renal failure, ARF – Acute renal failure, DM - Diabetes mellitus, HTN - Hypertension, IHD - Ischemic heart disease, FUO - Fever of unknown origin

Table 2 shows the age- and sex-wise distribution of VAP patients. The present study showed maximum number of cases (n = 31) were between 46 and 60 years of age, and people in this age group had the highest percentage of VAP (29.54%) also.

**Table 2:** Age- and sex-wise distribution of VAP and control (non-VAP) group of patients

Age(yrs)	Total patients (n=97)	VAP (n=44) (%)	Non-VAP (n=53) (%)
0–15	2	1 (2.27)	1 (1.89)
16–30	15	6 (13.64)	9 (16.98)
31–45	26	12 (27.27)	14 (26.42)
46–60	31	13 (29.54)	18 (33.96)
>60	23	12 (27.27)	11 (20.75)
Sex			
Male	69	33 (75)	39 (67.9)
Female	28	11 (25)	17 (32.1)

### Risk factor analysis

As seen in Table 3, all data were analyzed by using Fisher's exact test. Reintubation *(P*<0.0001) and stress ulcer prophylaxis (*P*<0.05) were found to be the significant risk factors associated with the development of VAP. Although many studies have shown that previous antibiotic treatment is a significant risk factor for development of VAP, yet our study does not support it (*P*=0.369). In other words, as the number of patients in this respect is very less in our study, it is difficult to comment about the statistical significance of this risk factor. Our study also shows that although maximum number of patients were from postsurgical ward in our MICU setup, yet surgery is not a risk factor for VAP (*P*=0.825).

**Table 3:** Risk factors for VAP in this study

Factors	VAP (n=44)	Non-VAP (n=53)	*P*-value
Re-intubations	14	1	*P*<0.0001
Use of broad spectrum antibiotics (preceding 7 days)	7	5	*P*<0.369
Surgery	21	22	*P*<0.825
Stress ulcer prophylaxis	22	13	*P*<0.05

Table 4 shows co-morbid factors like chronic renal failure, diabetes mellitus malignancy are not significant (*P*=0.418) in the process of causing VAP, but our study shows early, planned tracheostomy (7-8 days after starting of mechanical ventilation) reduces the chance of getting VAP (*P*<0.05).

**Table 4:** Patient-related factors in proces of VAP

Factors	VAP	Non-VAP	*P*-value
Co-morbid factors (n=28) (CRF, HTN, DM, Malignancy)	15	13	*P*=0.418
Early Tracheostomy (n=13)	2	11	*P*<0.05

**Figure 1:**
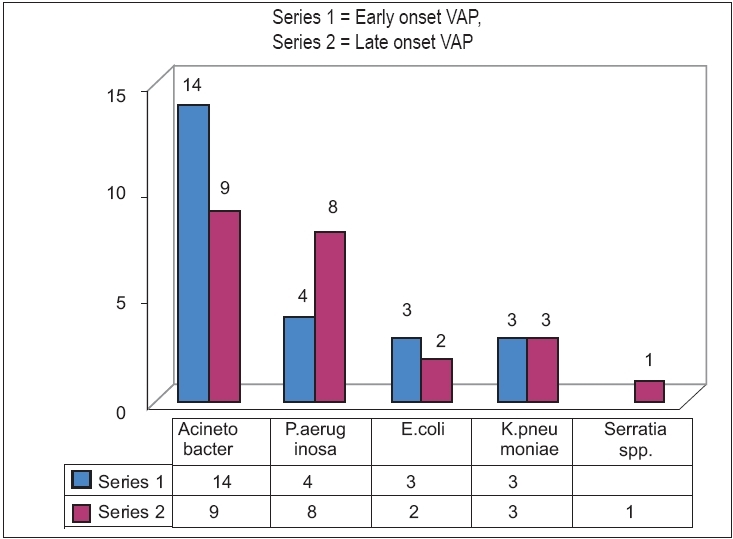
Organisms causing VAP (early- and late-onset VAP)

Figure 1 shows that *Acinetobacter* species is the commonest (48.94%) organism causing early-onset and late-onset VAP in our setup, followed by *Pseudomonas aeruginosa* (25.53%). Other common organisms are *Escherichia coli* (10.64%), *Klebsiella pneumoniae* (12.77%) and *Serratia marcescens* (2.13%).

**Figure 2:**
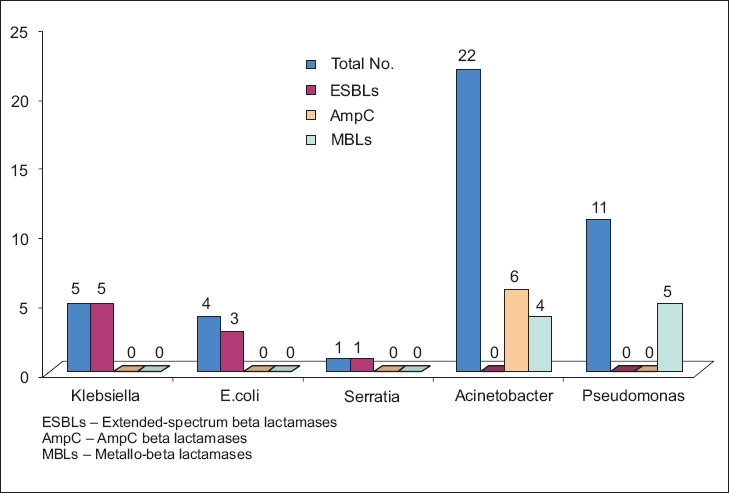
Different enzymes produced by the isolated strains. ESBLs - Extendedspectrum beta lactamases, AmpC - AmpC beta lactamases, MBLs - Metallo-beta lactamases

Multidrug-resistant (MDR) organisms are a major threat in our MICU setup. Table 5 shows that among the 23 isolates of *Acinetobacter* species, 5 (21.74%) were resistant to all groups of antibiotics, including carbapenems. Among the 12 isolates of *Pseudomonas aeruginosa*, 6 (50%) were resistant to all groups of antibiotics, even carbapenems. All the above carbapenem-resistant strains were MBLs-producing strains [Diagram 2. Other potential multidrug-resistant organisms like *E. coli*,*K. pneumoniae* and *S. marcescens* were all ESBLs-producing strains. Among the 23 strains of *Acinetobacter* spp, 7 (30.43%) showed AmpC beta lactamase producing strains [Figure 2].

**Table 5:** Antibiotic Sensitivity pattern of the isolates

Organism	No. of isolates	Sensitive	Resistant
Acinetobacter *spp.*	23	NET (15), CFS (18), AMI (4) TZP (5), IMI (14), MEM (14)	IMI and MEM (5), AMI (15)
Ps. aeruginosa	12	AMI (2), TZP (6), IMI (6), MEM (6), ATZ (3) CTZ (4)	IMI and MEM (6), AMI (10), CTZ (8), ATZ (3)
Kleb.pneumoniae	6	NET (4), AMI (4), CFS (6), TZP (5), IMI (6), MEM (6)	AMP (6), GEN (4), CIP (4), CTX (6)
*E.* coli	5	NET (5), AMI (5), CFS (5), IMI (5), MEM (5)	GEN (3), CIP (3) CTX (5), ATZ (5)
S. marcescens	1	CFS, TZP, IMI, MEM	GEN, AMI, CIP, CTX, ATZ

GEN - Gentamicin, NET - Netilmycin, AMI - Amikacin, CIP - Ciprofloxacin, ATZ - Aztreonam, CTX - Cefotaxime, CTZ - Ceflazidiem, CFS - Cefoperazone - Sulbactam, TZP - Pipercillin-Tazobactam, IMI - Imipenem, MEM - Meropenem.

## Discussion

This study demonstrated that VAP is an important nosocomial infection among patients receiving MV in MICU of our tertiary care hospital. The risk of VAP is highest early in the course of hospital stay and is estimated to be approximately 3% per day during the first 5 days of ventilation and approximately 2% per day during days 5 to 10 of ventilation[8]; thereafter, it is essential to have a high degree of suspicion of VAP in the first week after intubation. The incidence of VAP in our study was 45.4%, which is bit higher than other previous studies.[13,15,20] But the study by Rajasekhar *et al.*[10] shows the incidence was much higher and the number of study people was also less in their study. The total number of cases in our study and the study duration also was less comparatively, which may reflect the higher incidence of VAP in our MICU. The higher incidence of VAP in our study may be due to co-morbid conditions of our patients. Mostly, the patients came from distant hospitals to our tertiary care setup at a terminal stage, with malignancy, leptospirosis or complicated malaria, etc. These terminal conditions of the patients demand more number of days on mechanical ventilation.

Our study shows patients of age more than 30 years are more prone to get VAP on prolonged MV, and it also shows that gender has no significant role in the development of VAP [Table 3].

Reintubation is a definitive risk factor for VAP that has been shown previously by many studies, and our study also shows the significance of that risk factor causing VAP (*P*<0.0001).

Although stress ulcer prophylaxis is a controversial factor for VAP, we found that it is a significant risk factor in our setup (*P*<0.05) [Table 3]. Many studies have shown that stress ulcer prophylaxis is beneficial[20,21] for patients, but many studies have also shown that it is a risk factor for development of VAP.[22,23] The use of antacids in patients with VAP suggests that colonization of the stomach with pathogenic bacteria may have contributed to the occurrence of VAP.

Co-morbid factors are not very significant in our study, but it shows that early (7-8 days after starting of MV), planned tracheostomy is beneficial to the patient to prevent VAP, as shown by Panwar *et al.*[24] recently.

Multidrug-resistant organisms are increasing in our MICU setup. Earlier reports show that among the gram-negative organisms, *Pseudomonas aeruginosa* is the commonest causative agent of VAP,[4,11] but in the present study *Acinetobacter* spp. was found to be the commonest (48.94%) isolate, followed by *Pseudomonas aeruginosa* (25.53%).

Presently there is concern about the acquisition of plasmid-mediated metallo-beta lactamases active against carbapenems and antipseudomonal penicillins and cephalosporins.[25,26] Although *Acinetobacter* spp. are generally less virulent than *P. aeruginosa,* these have nonetheless become problem pathogens because of increasing resistance to commonly used antimicrobial agents.[27] More than 85% of isolates are susceptible to carbapenems, but resistance is increasing due either to IMP-type metalloenzymes or carbapenemases of the OXA type.[26] In our study, six isolates of *Pseudomonas* and five isolates of *Acinetobacter* spp. were plasmid-mediated metallo-beta lactamases enzyme producing strains, detected by imipenem-EDTA disk method.[19]

Extended-spectrum beta lactamases (ESBLs) and AmpC beta lactamases are of increasing clinical concern. ESBLs are most commonly produced by *Klebsiella* spp. and *Escherichia coli* but may also occur in other gram-negative bacteria. They are typically plasmid-mediated clavulanate susceptible enzymes that hydrolyze penicillins, expanded-spectrum cephalosporins (cefotaxime, ceftriaxone, ceftazidime, cefepime and others) and aztreonam. AmpC beta lactamases are cephalosporinases that are poorly inhibited by clavulanic acid. They can be differentiated from other ESBLs by their ability to hydrolyze cephamycins (Cefoxitin, Cefotetan) as well as other extended-spectrum cephalosporins. AmpC beta lactamases, demonstrated to be chromosomally or plasmid mediated, have been described in pathogens, e.g., *Klebsiella pneumoniae*, *Escherichia coli*, *Salmonella* spp., *Proteus mirabilis, Citrobacter freundii*, *Acinetobacter* spp., *Enterobacter* spp. and *Pseudomonas aeruginosa.*

Although the current Clinical Laboratory Standards Institute (CLSI) guidelines do not describe any method for detection of isolates producing AmpC beta lactamases, we followed the popular AmpC-disk method[18] to detect AmpC beta lactamases among our isolates. Seven (30.43%) out of 23 isolates of *Acinetobacter* spp. have shown production of AmpC beta lactamase enzyme, and no other Enterobacteriaceae showed production of AmpC.

Many studies have shown that methicillin-sensitive *Staphylococcus aureus* (MSSA) or methicillin-resistant *Staphylococcus aureus* (MRSA) are major causative agents of early-onset VAP,[8,11] but we did not get a single isolate of *Staphylococcus aureus* in our setup. This finding indicates that the causative pathogens always vary in different setups. This finding will also help as an epidemiological marker for initial prophylactic treatment planning for mechanically ventilated patients in our MICU setup.

We did not get any anaerobic organism as VAP pathogen, which corroborates with earlier study findings[28] also and signifies that anaerobes are not the usual pathogens causing VAP. Fungal pathogens are also not significant agents causing VAP.[29,30] Among our cases, we isolated *Candida albicans* from one elderly longstanding diabetic patient, and the colony count was also very less (<10^3^ cfu/ml), which determines that *Candida* was a tracheal colonizer only.

Although for the diagnosis of VAP we did quantitative culture of ETA (≥10^5^ cfu/ml) and corroborated other findings with CPIS scoring (CPIS >6), yet the results from this study need to be validated by comparison to gold standards such as histology of lung tissue.

This study showed that quantitative culture of ETA is a useful test for early diagnosis of VAP and also helped to determine the incidence of MDR organisms causing VAP in our MICU setup. The antibiotic susceptibility pattern of these isolates will also help the clinicians to choose the appropriate antimicrobial agents for prophylactic as well as treatment purposes. This study revealed differences in VAP incidence in our MICU and literature data, emphasizing judicious use of antimicrobial therapy. Combined approaches of rotational antibiotic therapy and education programs might be beneficial to combat high antibiotic resistance in our setup.
